# Pre-Clinical Models of Traumatic Brain Injury—A Narrative Review Towards “Animal Neuro-ICUs”

**DOI:** 10.3390/biomedicines14030688

**Published:** 2026-03-17

**Authors:** Franziska Münz, Andrea Hoffmann, Michael Gröger, Ohad Sharon, Magnus Scheer, Sandra Kress, Maximilian Feth, Peter Radermacher, Thomas Kapapa

**Affiliations:** 1 Institute for Anesthesiological Pathophysiology and Process Engineering, University Ulm, 89081 Ulm, Germanypeter.radermacher@uni-ulm.de (P.R.); 2 Department of Anesthesiology and Intensive Care Medicine, University Hospital Ulm, 89081 Ulm, Germany; 3 Department of Neurosurgery, University Hospital Ulm, 89081 Ulm, Germanythomas.kapapa@uniklinik-ulm.de (T.K.); 4 Department of Neurosurgery, German Federal Armed Forces Hospital Ulm, 89081 Ulm, Germany; 5 Department of Anesthesiology, Intensive Care Medicine, Emergency Medicine and Pain Therapy, German Federal Armed Forces Hospital Ulm, 89081 Ulm, Germany; 6 Department of Intensive Care and Hyperbaric Medicine, University Hospital Angers, 49100 Angers, France

**Keywords:** neurocritical care, animal models, comorbidities, sex

## Abstract

The presence of traumatic brain injury (TBI) is a critical determinant of post-traumatic mortality and morbidity. Not only is TBI one of the leading causes of death among severely injured patients, but it also substantially impacts long-term outcomes following severe trauma. Neurocritical care has a profound effect on outcomes following brain injury; nevertheless, its application in preclinical studies remains infrequent. This review therefore discusses strategies to improve the translational relevance of experimental TBI research, including the integration of neurocritical care principles in animal models. The review further addresses the impact of observation periods after injury and the selection of appropriate animal models (large vs. small animal models). In addition, commonly used injury induction methods—including controlled cortical impact (CCI), fluid percussion injury (FPI), weight-drop models, and blast injury paradigms—are discussed in terms of their reproducibility and clinical relevance. Finally, the review explores whether age, comorbidities, and sex influence TBI outcomes—and, if so, how these variables should be incorporated into experimental designs to improve translational fidelity.

## 1. Introduction

Among patients with severe trauma, traumatic brain injury (TBI) has been identified as one of the leading causes of death [1], accounting for up to 58% of trauma-related fatalities in retrospective analyses [2]. Beyond its impact on early survival, TBI substantially affects functional and neurological recovery, thereby contributing to long-term disability [3].

In clinical neurocritical care, the management of severe TBI is guided by international recommendations, including those of the Brain Trauma Foundation [4]. Standard therapies include sedation, osmotherapy, and ventilation strategies, all aimed at controlling intracranial pressure (ICP) and maintaining adequate cerebral perfusion pressure (CPP) [5], defined as the difference between mean arterial pressure (MAP) and intracranial pressure (CPP = MAP − ICP). Continuous ICP monitoring has become an essential component of contemporary TBI care. In contrast, more advanced techniques such as cerebral microdialysis or brain tissue oxygenation (PbtO_2_) measurement are not part of routine monitoring in all hospitals.

Despite these advances in monitoring and supportive care, a major challenge persists: the translation of preclinical findings into clinically effective therapies. Experimental models are essential, as they provide important insights into pathophysiological mechanisms and allow the testing of potential therapeutic interventions prior to clinical application. However, numerous experimental interventions that proved beneficial in animal models have failed in clinical trials [6]. This translational gap reflects, in part, the limited capacity of conventional animal models to reproduce the multifactorial nature of human TBI, including variability in injury mechanisms, demographic factors and comorbidities [6]. Another important factor may be the discrepancy in the duration of post-injury monitoring. While patients with moderate-to-severe TBI typically require prolonged neurocritical care, experimental studies often investigate only short observation periods.

Literature Search and Selection: This narrative review is based on a targeted literature search conducted in PubMed. Relevant publications addressing experimental models of traumatic brain injury, neurocritical care concepts, and translational aspects of preclinical research were identified and evaluated. Additional studies were identified through manual screening of reference lists of relevant articles. The selection of literature was guided by relevance to the scope of this review, with priority given to original experimental studies, translational research, and recent review articles.

## 2. Translational Research in TBI

The aim of translational research is to convert findings from experimental models into effective clinical interventions. In the context of TBI, however, the majority of experimental strategies have failed to show benefit in phase II or III clinical trials. A well-documented example is progesterone, which exhibited neuroprotective effects in rat models through reduction in inflammation, prevention of neuronal loss, and improvement in functional outcomes [7,8]. Even, initial clinical findings also appeared promising: in the ProTECT II trial, a single-center phase II study, progesterone was administered intravenously within 4 h of injury and continued for 96 h. The trial reported reduced mortality in patients with severe TBI as well as improved Glasgow Outcome Scale scores in moderate TBI patients at 30 days post-injury [9]. However, these early results were not replicated in the subsequent large-scale, multicenter phase III ProTECT III and SyNAPSe trials, in which progesterone was administered within 4 to 8 h of injury for 96 to 120 h, respectively; no significant clinical benefit was observed at 6 months post-injury [10,11]. The progesterone trials illustrate the persistent translational gap between preclinical and clinical TBI research. While rodent models demonstrated robust neuroprotective effects under controlled experimental conditions, clinical outcomes diverged substantially. The ProTECT trial suggested short-term benefit at 30 days, but the larger and methodologically more rigorous SyNAPSe trial failed to confirm efficacy at 6 months. These discrepancies underscore how differences in species, timing of administration, treatment duration, and endpoint selection can critically influence translational success. Similar translational challenges have been observed for other therapeutic strategies investigated in TBI. For example, NMDA receptor antagonists [12] and magnesium sulfate [13] demonstrated neuroprotective effects in experimental models but failed to show consistent clinical benefit in subsequent human trials.

One of the most important limitations of current preclinical models of TBI is the insufficient integration of neurocritical care elements that are standard in clinical practice—particularly continuous monitoring of ICP and CPP. Clinical studies and meta-analyses have shown that multimodal monitoring, especially when PbtO_2_-guided therapy is used alongside ICP/CPP management, may improve survival, whereas findings on neurological outcome have been inconsistent [14,15]. However, these practices remain largely absent in experimental models, especially in rodents.

The implementation of continuous monitoring in preclinical models may be impeded by several critical factors, such as the technical challenges of adapting neuromonitoring devices to small animal anatomy, limited personnel resources, and the substantial financial burden associated with advanced neuromonitoring equipment [16,17]. In fact, the lack of standardized neurocritical care protocols in animal models—including the absence of ICP monitoring, controlled ventilation, and sedation—has been recognized as a major obstacle to improving translational relevance.

Nevertheless, several experimental studies have demonstrated that elements of neurocritical care can be implemented in animal models of traumatic brain injury. In murine models, Blasiole et al. [18] and Zusman et al. [19] developed combined paradigms of controlled cortical impact (CCI) and hemorrhagic shock that incorporated monitoring of systemic physiological parameters such as MAP and arterial blood gases, together with structured resuscitation strategies including targeted fluid administration, blood transfusion, and controlled oxygen therapy. In large animal models, particularly in swine, several groups have implemented experimental paradigms that more closely resemble clinical neurocritical care management. Datzmann et al. developed a long-term resuscitated porcine model of acute subdural hematoma (ASDH) that incorporated mechanical ventilation, fluid resuscitation, and vasopressor-supported hemodynamic stabilization to maintain CPP. Continuous multimodal neuromonitoring—including measurements of ICP, CPP, and PbtO_2_—was combined with repeated neurological assessment and prolonged observation periods exceeding 50 h [20]. Similarly, O’Donnell et al. described the establishment of a dedicated experimental neuro-intensive care unit for swine with TBI or subarachnoid hemorrhage. Their platform integrates multimodal neuromonitoring techniques such as ICP monitoring, PbtO_2_ measurement, electroencephalography (EEG), and cerebral microdialysis with mechanical ventilation, sedation strategies, and targeted physiological management [21].

As emphasized by O’Donnell et al., “Neurocritical care significantly impacts outcomes after brain injury, but is rarely applied in preclinical studies… Incorporating neurocritical care will reduce the translational gap for therapeutics and diagnostics specifically tailored for moderate-to-severe acquired brain injury”. Addressing these limitations requires a paradigm shift in experimental design, one that aligns preclinical models more closely with the complex realities of neurocritical care and, thereby, hopefully facilitates the development of interventions with genuine clinical potential.

## 3. Critical Care Animal Models for TBI

Rodent models remain the most widely used in experimental TBI research due to their cost-effectiveness, practical feasibility, availability of multiple test kits and limited need for post-operative care. Despite their widespread use, there are fundamental differences in neuronal anatomy, physiology, and behavior that limit the translational applicability of rodent models, as these characteristics are not representative of the human condition [22,23,24,25].

**Figure 1:**
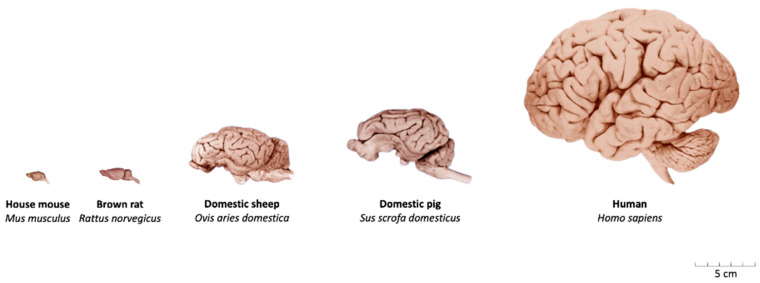
Overview of the macroscopic neuroanatomy of various species. Images reproduced and adapted from the Comparative Mammalian Brain Collections of the University of Wisconsin and Michigan State, and from the National Museum of Health and Medicine (https://brainmuseum.org/index.html, accessed on 10 November 2025).

An important consideration in translational TBI modeling is the surface anatomy of the brain (Figure 1 illustrates the major neuroanatomical differences between lissencephalic and gyrencephalic species).

**Figure 2:**
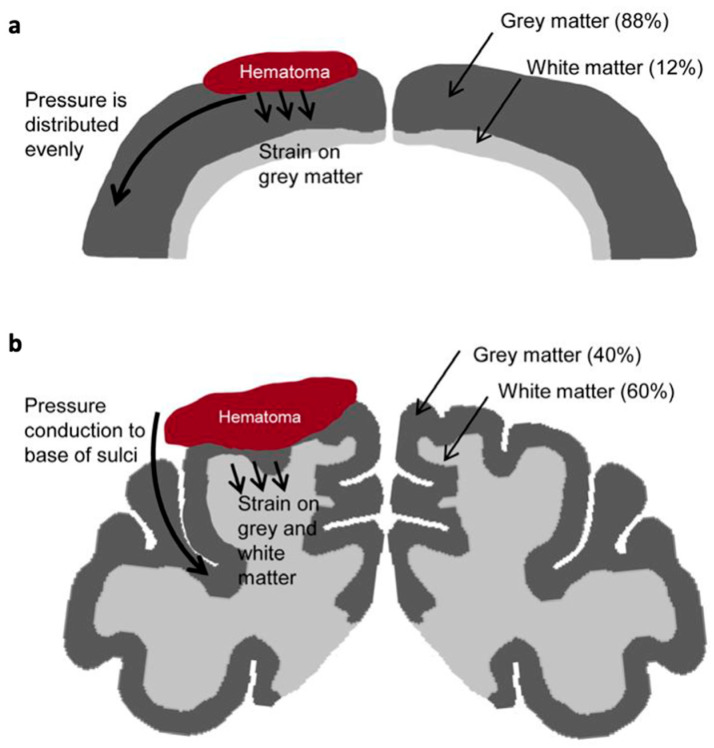
Schematic representation of pressure distribution in lissencephalic and gyrencephalic brains following acute subdural hematoma (ASDH). (**a**) In the lissencephalic brain, ASDH-induced pressure is distributed uniformly across the cortical surface. (**b**) In contrast, in the gyrencephalic brain, pressure propagation follows the contours of the sulci, resulting in maximal stress at their bases where vascular supply is located. Reprinted from McCook O et al. Localization of the hydrogen sulfide and oxytocin systems at the depth of the sulci in a porcine model of acute subdural hematoma [28]; open access article distributed under the Creative Commons Attribution-NonCommercial-ShareAlike 4.0 License (CC BY-NC-SA 4.0) (https://creativecommons.org/licenses/by-nc-sa/4.0/, accessed on 13 November 2025).

The lissencephalic structure of the rodent brain renders it suboptimal for extrapolation to the human brain, which is gyrencephalic. In lissencephalic species, mechanical stress following traumatic impact is distributed more uniformly and, hence, concentrated near the cortical surface. In contrast, in gyrencephalic brains—such as those of primates and humans—maximum mechanical stress is redirected toward deeper regions, particularly at the bases of the sulci (Figure 2) [21,26,27,28].

Furthermore, the white-to-gray matter ratio plays a critical role in the biomechanical and pathophysiological response to TBI. White matter, being more susceptible to edema [29,30,31], is a major determinant of pressure-induced secondary injury. The rodent brain consists of only 12–14% white matter, compared to approximately 60% in the human brain [32]. This fundamental difference suggests that the progression and localization of injury-related swelling and ICP in rodent models may not accurately reflect the human condition.

Another important anatomical difference concerns the structure of the skull and intracranial compartmentalization. In humans and larger, higher developed animal species, the tentorium cerebelli is a rigid dural fold that effectively separates the cerebrum from the cerebellum and constrains the distribution of injury-induced edema and elevated ICP to the supratentorial compartment [33,34]. Rodents, by contrast, possess only a vestigial, pliable connective tissue membrane in place of a rigid tentorium. As a result, increases in cerebral pressure following injury can redistribute more freely across intracranial compartments in rodents, whereas in humans, compartmentalized pressure elevation can lead to region-specific pathophysiological consequences [34].

Taken together, these anatomical and structural disparities highlight the limitations of rodent models in accurately reproducing critical pathophysiological features of human traumatic brain injury.

Large animal models, particularly in swine, offer a high degree of anatomical and physiological similarity to humans, including gyrencephalic brains, comparable white-to-gray matter ratios, and the ability to utilize standard clinical monitoring equipment [21,28]. The gyrencephalic architecture of the porcine brain facilitates injury responses that involve both gray and white matter compartments, thereby more closely recapitulating the tissue-level pathophysiology observed in humans. Moreover, the cerebrovascular anatomy of the pig—particularly with respect to the localization and distribution of cortical surface vessels—shares notable similarities with that of the human brain. Nevertheless, the presence of a carotid rete mirabile instead of a classical Circle of Willis represents a species-specific difference that primarily affects proximal arterial inflow, while distal cortical perfusion patterns remain comparable. The presence of gyri and sulci further influences the propagation and focal concentration of mechanical forces toward subcortical regions, enhancing the biomechanical fidelity of this species for TBI research [29,35].

An additional anatomical domain in which the pig demonstrates greater congruence with human neuroanatomy than rodents is the cranial architecture. This includes not only the morphology of the osseous skull but also the internal dural organization. In both pigs and humans, the presence of a rigid tentorium cerebelli contributes to anatomically distinct intracranial compartmentalization [29,31]. This similarity facilitates more accurate modeling of compartment-specific pathologies, such as transtentorial herniation and compartmentalized ICP elevation.

Finally, due to their size and bodyweight, human-sized models allow the use of standard clinical neuromonitoring equipment and neurocritical care protocols, including mechanical ventilation, multimodal neuromonitoring (ICP, PbtO_2_, temperature, microdialysis), and sedation management. Among these, swine models are most widely used because their gyrencephalic brain structure, cerebrovascular anatomy, and systemic physiology closely resemble those of humans, whereas ovine and primate models provide complementary advantages in specific contexts. For example, sheep—with their more favorable brain-to-body ratio and less pronounced neck musculature compared to pigs—are particularly suitable for impact acceleration-induced TBI, especially in juvenile animals, and have therefore been frequently used in acceleration injury studies [36]. Primate and ovine models are also advantageous for studies requiring complex behavioral assessments [37,38]. These features make large-animal models particularly suitable for investigating moderate-to-severe TBI under conditions that approximate human intensive care, thereby improving the translational relevance of experimental findings.

Both rodent and large-animal models therefore serve complementary roles in experimental TBI research. While rodent models remain indispensable for mechanistic and genetic investigations, large-animal models provide unique opportunities for studying systemic physiology and neurocritical care interventions under clinically relevant conditions. Table 1 summarizes key considerations that may guide the selection of rodent versus large-animal models depending on the specific research objective.

## 4. Induction Methods in Experimental TBI

The choice of injury induction technique critically shapes the pathophysiological trajectory and translational relevance. Each method has specific strengths and limitations regarding reproducibility and clinical fidelity.

### 4.1. Blast Injury Models

**Figure 3:**
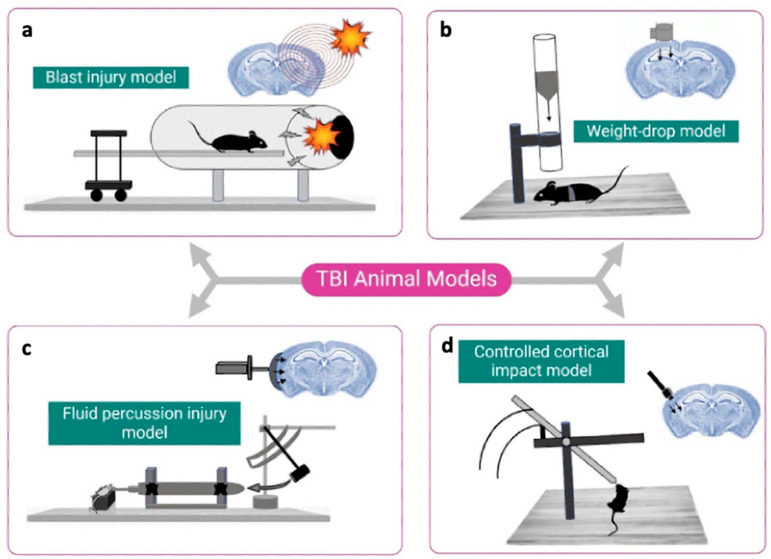
Overview of various animal models of TBI. Reprinted from Chauhan P et al. Animal Models of Traumatic Brain Injury and Their Relevance in Clinical Settings [39]. (**a**) Blast injury model, (**b**) Weight-drop model, (**c**) Fluid percussion injury model and (**d**) Controlled cortical impact model. Reprinted under the terms of the Creative Commons Attribution 3.0 License (CC BY 3.0) (https://creativecommons.org/licenses/by/3.0/, accessed on 8 July 2025). No changes were made to the original figure.

Blast injury models (Figure 3a) have been developed to study blast-related mechanisms of traumatic brain injury, which are particularly relevant in military settings [39,40,41]. These models typically use shock tubes or controlled gas detonations to expose animals to a single, well-defined overpressure wave. The resulting biomechanical forces lead to rapid deformation of the skull, fluid shifts, and pressure gradients across the brain parenchyma. Pathophysiologically, blast exposure induces a range of injury features observed in human TBI, including diffuse axonal injury and blood–brain barrier disruption [39,42,43]. A major strength of blast models lies in their capacity to isolate the effects of primary blast exposure, excluding confounding factors such as impact acceleration or secondary shrapnel trauma. This makes them particularly useful for investigating mechanistic questions. However, despite these advantages, blast models face substantial limitations in terms of standardization and translational value. The complexity of replicating real-world blast scenarios has led to wide inter-laboratory variability in injury parameters, including tube design, animal positioning, and exposure profile. Moreover, many secondary factors present in human blast injuries—such as thermal burns, polytrauma, and hypoxemia [39]—are not captured in experimental paradigms.

In summary, blast injury models are best suited for exploring the distinct biomechanical and neurobiological consequences of primary overpressure exposure. While they offer unique insights into blast-specific pathomechanisms, their clinical translation is constrained by methodological heterogeneity and limited overlap with common civilian TBI phenotypes. As such, their use should be guided by focused research questions.

### 4.2. Weight-Drop Models

The weight-drop model (Figure 3b), particularly in the version developed by Marmarou et al., has long been employed to simulate closed-head injury and diffuse brain trauma in rodents [44,45]. The technique involves dropping a defined weight from a pre-determined height onto a metallic disc affixed to the skull, thereby transmitting mechanical energy through the cranium without the need for craniectomy. This feature has made the model attractive for simulating acceleration–deceleration injury mechanisms such as those encountered in falls, sports injuries, or motor vehicle accidents, where the skull remains intact [46,47]. Histopathologically, the Marmarou weight-drop model primarily induces diffuse axonal injury, although traumatic hemorrhages such as subarachnoid, intraparenchymal, or ASDH may also occur depending on impact severity and alignment [44]. However, the variability and limited control of biomechanical parameters—such as drop height and energy transmission—result in considerable inter-animal and inter-laboratory variability, which constrains reproducibility and thus translational applicability [39,48].

In summary, the Marmarou weight-drop model remains a valuable tool for inducing closed-head diffuse TBI, particularly in low-resource or exploratory settings. However, limited parameter control, high inter- and inter-laboratory variability, and the poor scalability of free-fall paradigms to gyrencephalic large-animal brains constrain its use in reproducible translational research. Careful model selection and stringent experimental standardization are therefore essential when employing weight-drop paradigms in TBI research.

### 4.3. Fluid Percussion Injury (FPI)

The FPI model (Figure 3c) remains one of the most widely used and well-characterized paradigms for preclinical TBI. A single rapid fluid pulse delivered after craniectomy [49] deforms the intact dura mater and in some cases the underlying cortex tissue, producing a transient mechanical insult. Two primary variants are distinguished by craniectomy location: midline FPI and lateral FPI, each offering distinct pathophysiological and translational features [50,51].

Midline FPI, in which the craniectomy is centered on the sagittal suture, induces a diffuse, bilateral brain injury in the absence of overt cavitation or contusion [52,53]. By contrast, lateral FPI, performed with a craniectomy over the parietal cortex, produces a combination of focal cortical contusion and diffuse injury [51,54]. Each variant has specific strengths and limitations. Midline FPI is reproducible and ideal for modeling diffuse mechanisms, biomarker dynamics (e.g., Glial Fibrillary Acidic Protein (GFAP), Ubiquitin Carboxy-Terminal Hydrolase L1 (UCH-L1)), and neurobehavioral trajectories without confounding focal pathology [50]. However, focal pathologies, including post-traumatic epilepsy, are more effectively modelled using lateral FPI, which induces pronounced cortical contusion and focal tissue injury [6,55,56]. In addition, lateral FPI captures a broader range of histopathological outcomes, but suffers from greater variability in lesion size due to surgical approach [57]. Both models require craniectomy, potentially introducing surgical artifacts that, again, necessitate rigorous sham controls.

In conclusion, FPI models—especially when selected and calibrated based on research questions—offer valuable tools to investigate acute and chronic consequences of TBI. Midline FPI is best suited for diffuse injury research and biomarker exploration, whereas lateral FPI is more appropriate for studies involving focal lesions.

### 4.4. Controlled Cortical Impact (CCI)

Initially developed to replicate brain injuries resulting from motor vehicle collisions, the CCI model (Figure 3d) has since become a well-established and widely adopted technique in experimental TBI research. The model employs a mechanically driven piston—typically actuated pneumatically or electromagnetically—that delivers a defined impact to the surgically exposed dura mater. Variations in the size, shape, and material of the impactor tip, as well as adjustable parameters such as velocity, depth, dwell time, and impact location, allow for a high degree of experimental control and inter-study comparability across species [58,59] and injury severities.

CCI reliably induces morphological and cerebrovascular alterations that reflect several hallmarks of human focal TBI, including cortical contusion [60,61], blood–brain barrier disruption [62,63], subdural and intraparenchymal hematoma formation, vasogenic edema, inflammation [64], and impaired blood flow to the brain [65]. These pathologies are accompanied by neurobehavioral and cognitive deficits [59,66]. Despite these strengths, the CCI model predominantly generates focal contusional injuries and does not adequately recapitulate the diffuse axonal and multifocal injury patterns characteristic of many clinical TBI cases [67]. Moreover, the requirement for craniectomy introduces surgical trauma that, although biomechanically negligible at the tissue level, may confound interpretation of neuroinflammatory or vascular endpoints [67]. Therefore, rigorous experimental design necessitates the inclusion of appropriate surgical sham controls [68]. Importantly, adaptations of the CCI model now permit closed-skull impacts, enabling investigation of mild and repetitive TBI under more clinically analogous conditions.

### 4.5. Acute Subdural Hematoma

ASDH represents a common form of traumatic brain injury. In experimental research, ASDH serves as a well-reproducible model of focal mass-effect injury. Foundational work in rats demonstrated that subdural blood accumulation consistently produces a sharply demarcated zone of cortical ischemia beneath the hematoma, allowing the inference of a causal link between mass effect and ischemic injury in experimental ASDH models [69].

**Figure 4:**
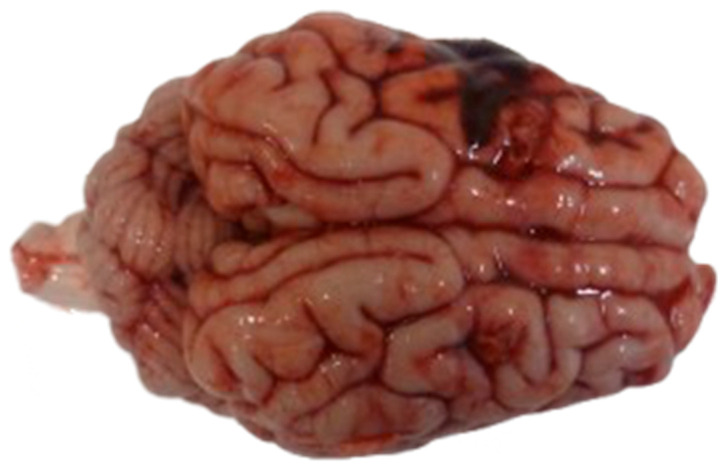
Macroscopic view of a porcine brain immediately after termination of the experiment, demonstrating a subdural hematoma over the left hemisphere.

In large-animal models, ASDH is generated by controlled deposition of autologous blood into the subdural compartment to reproduce the biomechanical and physiological sequelae of a hematoma. After surgical exposure, a craniotomy over the parietal cortex allows placement of a subdural catheter through which autologous blood is infused [20], thereby creating a well-defined, space-occupying lesion (Figure 4). The injected volume typically corresponds to 10–15% of total brain volume, reflecting the supratentorial threshold of volume tolerance identified in previous porcine studies [70,71]. Using this approach, Timaru-Kast et al. demonstrated dose-dependent increases in intracranial pressure, cerebral perfusion impairment, metabolic derangement, and histopathological injury in pigs, confirming the translational relevance and reproducibility of large-animal ASDH models under multimodal neuromonitoring conditions [72].

In addition, the ASDH model permits the integration of a systemic hemorrhage to emulate polytrauma conditions frequently observed in severely injured TBI patients. Combining the intracranial mass lesion with controlled blood loss reproduces the interaction between intracranial hypertension, impaired cerebral perfusion, and systemic hypovolemia, thereby capturing a clinically important dimension of severe neurotrauma. While this combined ASDH–hemorrhage approach has been particularly well established in swine—where physiological regulation, cardiovascular responses, and intensive-care management closely parallel the human condition—it has also been adapted in rodent models.

Rodent models provide an essential experimental platform for investigating the ischemic and pressure-driven consequences of ASDH under genetically and mechanistically controlled conditions. The foundational rat model introduced by Miller et al. established that subdural blood accumulation produces a sharply demarcated zone of cortical ischemia directly beneath the hematoma, linking mechanical mass effect and ischemic cellular injury [69]. To expand the model into genetic research, Sasaki and Dunn adapted the paradigm for use in mice, demonstrating that graded subdural blood volumes generate dose-dependent ischemic lesions comparable to those observed in rats [73]. More recently, refinements of the traditional rat approach have aimed to reduce procedure-related cortical trauma and to improve model consistency. Xian et al. developed a modified technique employing an optimized burr-hole position and a fusiform gavage needle, resulting in more concentrated ipsilateral hematoma formation, fewer inadvertent cortical injuries, higher survival rates, and improved reproducibility compared with the classical Miller method [74]. These advancements address known limitations of earlier rodent models, particularly the challenge of operator-dependent variability and unintended cortical damage around the injection site.

**Figure 5:**
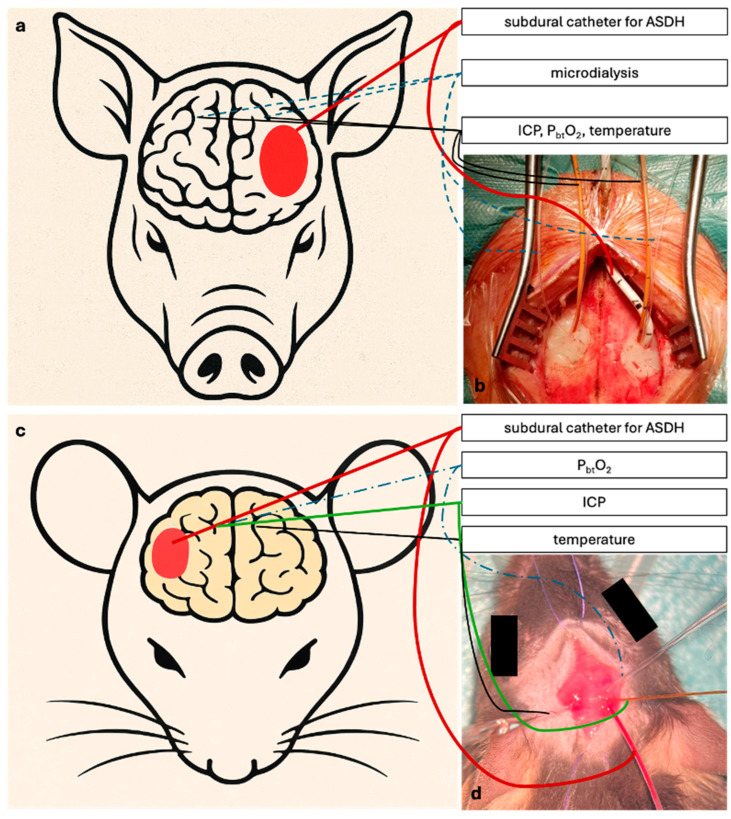
Experimental setup in swine and mouse. (**a**,**c**) Schematic illustrations of the experimental setup in swine (**a**) and mouse (**c**). (**b**,**d**) Representative photographs of the experimental setups in swine (**b**) and mouse (**d**).

The principal strength of the ASDH model lies in its reproducibility and its strong translational alignment with clinical neurotrauma. This is particularly true for large-animal preparations, in which the neurosurgical approach allows direct implantation of multimodal neuromonitoring probes and microdialysis catheters (Figure 5a). These models permit continuous measurement of intracranial pressure, cerebral perfusion pressure, tissue oxygenation, and cerebral metabolism under conditions that closely mirror human neurointensive care. As such, large-animal ASDH models uniquely capture the complex physiology, intracranial compliance, and systemic interactions characteristic of severe ASDH in patients, making them the most suitable platform for evaluating neurocritical care interventions and treatment strategies. Multimodal neuromonitoring can, in principle, also be implemented in rodent ASDH models (Figure 5b); however, this requires substantially increased technical effort, the use of size-adapted probes and catheters, and highly specialized surgical expertise. Consequently, while rodent models offer high experimental tractability and access to genetic manipulation and remain valuable for mechanistic studies, their ability to replicate comprehensive neurocritical care monitoring is inherently limited. Rodent ASDH models therefore serve primarily as complementary tools for mechanistic exploration, whereas large-animal models provide superior translational fidelity and are better suited for preclinical testing under clinically relevant conditions.

A notable limitation, however, is the requirement for burrhole approach, which—although essential for model establishment—introduces the potential for surgical artifacts. Despite this constraint, the ASDH paradigm remains a valuable component within the experimental TBI framework for investigating mass-effect-driven pathophysiology under conditions approximating clinical neurointensive care.

### 4.6. Reproducibility and Translational Relevance of TBI Induction Models

Among the established TBI induction models, reproducibility and translational value differ considerably. Weight-drop techniques are simple but highly variable and not suitable for large animals. Fluid percussion provides mixed injuries but requires craniotomy and is rarely scaled beyond rodents. Controlled cortical impact offers the best reproducibility, precise biomechanical control, and has been successfully applied in pigs, though it primarily induces focal contusions. Blast injury models, on the other hand, replicate the complex overpressure dynamics of military and civilian blast exposures and have been adapted to large animals, but they require specialized setups and still face challenges regarding reproducibility across laboratories. The ASDH model, in contrast, represents a highly reproducible paradigm of space-occupying focal injury. Overall, reproducibility in large-animal research is best achieved with controlled cortical impact, blast injury models and ASDH models, while weight-drop and fluid percussion remain mainly rodent-based approaches.

To provide a concise overview of key characteristics of commonly used TBI induction paradigms, Table 2 summarizes major models with respect to reproducibility, translational relevance, and compatibility with ICU-like monitoring approaches.

## 5. Length of Stay at the ICU After TBI

In clinical neurocritical care, patients with moderate-to-severe TBI frequently require extended stays in the intensive care unit (ICU). In some cases, intensive care unit stays have been reported to extend markedly beyond three weeks [75]. In contrast, the duration of post-injury monitoring in preclinical TBI studies typically ranges from no intensive care observation [76,77], to only a few hours [78,79,80,81], with extended monitoring remaining the exception [20,21,82,83] (Table 3). This mismatch between the length of clinical ICU care and the abbreviated observation periods in animal studies may critically hinder translational validity. Key pathophysiological processes—including delayed cerebral edema, neuroinflammation, excitotoxicity, and post-traumatic vasospasm—often evolve over days to weeks. Their dynamics influence treatment response, prognosis, and long-term neuroplasticity. Short monitoring windows in animal studies preclude adequate evaluation of these time-dependent events and may result in overestimation of early treatment efficacy or failure to detect delayed therapeutic effects. Moreover, the lack of extended ICU-like conditions in animal models prevents assessment of intermediate and long-term outcomes, including lesion maturation, behavioral recovery, delayed seizures, or therapy-induced structural plasticity. This limits the relevance of many preclinical endpoints for predicting clinical efficacy.

This discrepancy can be attributed to several factors, including the high costs associated with extended animal care, restricted personnel resources, and ethical concerns regarding the long-term use of intensive care procedures [21]. Despite these constraints, the abbreviated duration of observation in most animal studies is recognized as a limiting factor in preclinical TBI research [84], and study designs should aim to reflect not only the injury mechanism, but also the temporal complexity of human TBI care. Consequently, future studies should therefore strive to implement prolonged post-injury monitoring periods. This would allow for the characterization of evolving secondary injury mechanisms and provide more reliable insight into the durability of treatment effects. Aligning the duration and complexity of animal observation with the realities of clinical ICU care is essential to enhance the predictive validity and translational success of experimental TBI therapies but requires enormous effort with regard to infrastructure and personnel. A similar issue has been recognized in sepsis research, where short-term rodent models without intensive care support failed to predict clinical outcomes. As highlighted by Angus and van der Poll, the discrepancy between simplified animal models and the complex, time-dependent course of human sepsis has limited translation [85]—a lesson equally applicable to TBI.

## 6. Influence of Comorbidities, Age and Sex

### 6.1. Comorbidities and Age

TBI is increasingly prevalent among vulnerable populations such as the elderly or patients with pre-existing medical conditions [86,87]. Advanced age and comorbidities significantly influence TBI outcomes, including increased mortality and impaired functional recovery [88]. Moreover, comorbidities such as cardiovascular disease, diabetes, and psychiatric disorders exacerbate systemic inflammation and may compromise healing after injury [89]. Despite their relevance in clinical populations, comorbid conditions are largely absent from preclinical TBI models. Most experimental studies rely on young, otherwise healthy animals under standardized conditions, limiting external validity. However, emerging evidence suggests that comorbidities notably modulate outcomes even under controlled laboratory conditions. For instance, Datzmann et al. used a long-term resuscitated porcine model to investigate the effects of targeted hyperoxemia after TBI in pigs with [82] and without [83] coronary artery disease. The study demonstrated differences in both survival and neurological outcome, despite identical injury parameters and treatment protocols [82,83]. These findings highlight that pre-existing pathology can alter injury trajectories and treatment responses and thus should be accounted for in translational research models.

Moreover, age remains an underrepresented variable in preclinical TBI research, despite its well-established relevance in clinical settings. The majority of rodent models utilize animals in early adulthood, typically between 8 and 12 weeks of age, corresponding to human adolescence or young adulthood. Aged animals—those equivalent to middle-aged or elderly humans—are rarely included, although their physiological and pathological responses to traumatic brain injury differ markedly. In contrast, sepsis research has increasingly recognized the importance of age as a biological variable: studies using aged animals demonstrated altered immune responses, increased organ dysfunction, and higher mortality compared to young counterparts [90]. These findings underscore that incorporating age into experimental design can reveal clinically relevant disease mechanisms—an approach that should equally be adopted in TBI research. Incorporating aged animals into experimental models presents logistical and economic challenges, including higher mortality, increased biological variability, and a greater need for perioperative care. However, given the demographic reality of TBI in clinical populations—where the proportion of elderly patients is steadily increasing—the failure to adequately model age constitutes a major limitation of current translational strategies and should be explicitly addressed in future preclinical TBI models.

### 6.2. Sex

Biological sex is a relevant yet often underappreciated variable influencing both the incidence and outcome of TBI. Recent epidemiological analyses confirm that men continue to experience a higher incidence of traumatic brain injury than women across most age groups; however, this sex difference decreases with advancing age and may even reverse in older populations, where falls represent the predominant injury mechanism [91,92]. Sex-specific outcome differences after TBI have been observed across numerous clinical studies. Among patients with mild-to-moderate TBI, a majority of studies report worse outcomes in women, particularly with respect to post-concussive symptoms such as fatigue, dizziness, depression, and cognitive impairment. In contrast, studies investigating moderate-to-severe TBI often find better functional recovery in women compared to men [93]. These disparities may reflect a complex interplay between sex and injury severity as well as differences in the biological and psychosocial response to injury. Despite this clinical relevance, the majority of preclinical TBI studies continue to rely predominantly on young, healthy, male animals. For instance, a review found that 93–95% of preclinical TBI studies failed to include sex as a biological variable [94]. Moreover, a substantial number of studies either omit reporting the sex of animals altogether [81] or use castrated males [77,82,83] to reduce aggression and simplify housing, further distancing the experimental model from clinical physiology. This omission neglects potentially important sex-specific mechanisms of injury response and therapeutic efficacy. Where sex has been examined in animal models, outcomes have varied. Approximately 44% of studies reported better outcomes in female animals, while only 14% found worse outcomes, and the remainder showed either no difference or mixed results [93]. This heterogeneity may stem from differences in injury model, species, outcome measures, and hormonal status. In female animals, hormonal status may represent an additional source of biological variability. Factors such as the estrous cycle or hormonal fluctuations can influence injury responses and neurobiological processes following traumatic brain injury. Experimental evidence suggests that sex hormones, including estrogen and progesterone, may modulate inflammatory pathways [95] and brain edema formation after brain injury [96]. Consequently, hormonal status and cycle stage should be considered when designing experiments and interpreting results in studies involving female animals. Importantly, sex should not be treated as a confounder but as a biological variable that requires dedicated stratification and analysis.

The influence of comorbidities, age, and sex on TBI outcome is well established in the clinical setting but insufficiently modeled in preclinical research. Incorporating these variables—through the use of aged animals, disease models (e.g., cardiovascular comorbidity), and balanced sex representation—will improve the external validity and translational relevance of experimental studies. The choice of animal model should therefore be guided by the clinical phenotype being investigated, with attention to population-specific factors that shape injury evolution and therapeutic response.

## 7. Neuro-ICU for Animal Models

A major limitation of most preclinical TBI studies is the lack of intensive care measures that are routinely provided to patients with severe brain injury. In clinical neuro-ICU settings, continuous monitoring and organ support are integral to survival and recovery, whereas in animal models supportive care is often limited to anesthesia during injury induction and basic post-procedural surveillance. Establishing a “Neuro-Animal ICU” framework could therefore substantially increase translational validity. Key elements should include multimodal monitoring—encompassing ICP, CPP, PbtO_2_, microdialysis, and neurological assessment (e.g., modified Glasgow Coma Scale (MGCS)) as well as EEG—which are well established in human neurocritical care and have also been implemented in pig models [21,83,97]. Alongside neuromonitoring, maintenance of systemic homeostasis through controlled ventilation, fluid therapy, and vasopressor support is essential, yet remains inconsistently applied in animal experiments.

Importantly, several groups have made substantial efforts to approximate neurocritical care conditions in rodent models, particularly in mice subjected to controlled cortical impact combined with hemorrhagic shock. These models incorporate invasive arterial and venous catheterization, continuous blood pressure monitoring, intracranial pressure monitoring, and direct measurements of PbtO_2_ during defined shock, pre-hospital, and definitive care phases [19,98,99,100]. In addition, advanced experimental paradigms have enabled the impact of resuscitation strategies and oxygenation targets on cerebral physiology and neuronal survival [18,101,102]. However, despite their conceptual sophistication, current rodent Neuro-ICU models exhibit fundamental technical and biological limitations. Most notably, prolonged mechanical ventilation is rarely feasible in mice and rats. Consequently, clinically central variables such as ventilation strategies, controlled PaCO_2_ management, and long-term oxygen titration cannot be reliably reproduced [98,99]. Similarly, although fluid resuscitation and blood reinfusion protocols are well established, cardiovascular support remains limited. Continuous vasopressor or inotrope administration—standard practice in human neuro-ICUs to maintain cerebral perfusion pressure—cannot be stably implemented over extended periods in rodents, restricting precise CPP-guided therapy [19,100]. Another critical limitation is the short duration of intensive monitoring. Most murine Neuro-ICU studies focus on acute time windows ranging from several hours to, at most, 24–48 h after injury [19,98,99,100], whereas patients with severe TBI frequently require days to weeks of intensive care. This temporal mismatch limits the investigation of delayed secondary brain injury, systemic organ dysfunction, and ICU-associated complications. Moreover, while ICP and PbtO_2_ monitoring are technically feasible in rodents, these modalities are often applied in isolation or for limited time spans, rather than as part of a fully integrated, long-term multimodal neurocritical care strategy. In contrast, large-animal models permit comprehensive implementation of neurocritical care protocols.

A structured Neuro-Animal ICU also requires multimodal outcome assessment. This includes early neurological scoring, imaging (CT or MRI in large animals), metabolic monitoring (e.g., microdialysis), and blood or cerebrospinal fluid (CSF) biomarkers of neuronal and glial injury. The pig model has proven particularly valuable for such translational approaches, as shown by Kinder et al., who suggested that functional outcomes, biomarker profiling, and imaging are central to bridging experimental and clinical neurotrauma [103].

Clearly, implementing these protocols requires specialized infrastructure, trained personnel, and standardized operating procedures. However, the potential benefits—reduced variability, improved reproducibility, and enhanced translational value—outweigh the logistical challenges. A structured Neuro-Animal ICU therefore represents a crucial step toward bridging the gap between experimental TBI studies and clinical neurocritical care.

Even simplified implementations of a Neuro-Animal ICU may already enhance the physiological control and translational relevance of experimental TBI studies. At a minimum, this should include continuous monitoring of arterial blood pressure, oxygenation, body temperature, mechanical ventilation and fluid management, as well as measurement of ICP. More advanced modalities—such as PbtO_2_, cerebral microdialysis, or continuous EEG—may provide additional physiological insights but should be considered complementary depending on the available infrastructure and experimental objectives.

Indeed, any advancement in preclinical TBI models must also be aligned with the ethical framework of the 3R principles. The concept of a Neuro-Animal ICU can make an important contribution to refinement by integrating ICU-like monitoring and supportive care strategies into experimental models, thereby increasing the scientific value obtained from each individual animal experiment. For example, the use of controlled mechanical ventilation not only allows for deeper anesthesia, thus potentially reducing physiological stress due to attenuation of respiratory drive, but also yields more precise regulation of PaO_2_/PaCO_2_, i.e., major determinants of macrocirculatory cerebral blood flow and, thereby, ICP. In this way, more stable experimental conditions can be achieved, and clinically more relevant data can be generated. Although the implementation of Neuro-Animal ICU concepts may require increased financial and logistical resources, such models maximize the scientific benefit derived from each animal and thereby support both refinement and reduction within the framework of the 3R principles.

## 8. Conclusions

The development of structured, clinically oriented experimental platforms—so-called animal neuro-intensive care units—can represent a critical step toward improving translational fidelity in TBI research. By incorporating advanced monitoring modalities [20], extending the duration of observation [20], and including biological variables [82,83], such models would better reflect human injury trajectories and enable more meaningful evaluation of therapeutic interventions. Ultimately, aligning experimental conditions more closely with clinical neurocritical care practices seems to be essential to enhance the predictive validity of preclinical studies.

## Figures and Tables

**Table 1:** Model selection in experimental TBI research: considerations for rodent versus large-animal models.

Research Objective	Preferred Model	Rationale
Molecular and cellular mechanisms	Rodent models	Genetic tools and molecular techniques
Genetic pathways	Rodent models	Availability of transgenic models
Pharmacological screening	Rodent models	Efficient high-throughput testing
Systemic physiological responses	Large-animal models	Greater similarity to human physiology
Neurocritical care monitoring and interventions	Large-animal models	Enables ICU-level monitoring and support
Translational validation before clinical trials	Large-animal models	Higher anatomical and physiological comparability

**Table 2:** Comparison of commonly used experimental TBI models.

Model	Reproducibility	Translational Value	Compatibility with ICU-like Monitoring
Blast injury model	Limited due to inter-laboratory variability in injury parameters	Constrained by methodological heterogeneity and limited overlap with common civilian TBI phenotypes	Limited
Weight-drop model (Marmarou)	Variable due to limited control of biomechanical parameters	Limited translational applicability and poor scalability to large-animal brains	Limited
Fluid percussion injury (FPI)	Moderate–high reproducibility depending on model calibration	Moderate translational value; mixed focal and diffuse injury patterns	Limited in rodents
Controlled cortical impact (CCI)	High reproducibility and precise biomechanical control	Moderate translational relevance; primarily models focal contusions	Feasible in large animals
Acute subdural hematoma (ASDH)	Highly reproducible	Strong translational alignment with clinical neurotrauma	Highly compatible with multimodal neuromonitoring

**Table 3:** Examples of reported ICU-like monitoring duration, sex (♀: females; ♂: males), and age in neurotrauma research conducted in swine.

Authors, Year	Title	Duration of ICU-like Monitoring	Sex	Age/Weight
Wakam et al., 2021 [78]	“A single dose of valproic acid improves neurologic recovery and decreases brain lesion size in swine subjected to an isolated traumatic brain injury”	1 h	♀	12–16 weeks, 36–46 kg
Bambakidis et al., 2022 [79]	“Early Treatment With a Single Dose of Mesenchymal Stem Cell Derived Extracellular Vesicles Modulates the Brain Transcriptome to Create Neuroprotective Changes in a Porcine Model of Traumatic Brain Injury and Hemorrhagic Shock”	5 h	♀	12–15 weeks, 40–45 kg
Arnaud et al., 2022 [80]	“Effects of sequential aeromedical evacuations following traumatic brain injury in swine”	1.5 h	♂	10–12 weeks, 28–40 kg
Jin et al., 2023 [76]	“Prolonging the therapeutic window for valproic acid treatment in a swine model of traumatic brain injury and hemorrhagic shock”	-	♀	12–15 weeks, 40–45 kg
Forti et al., 2023 [81]	“Non-invasive diffuse optical monitoring of cerebral physiology in an adult swine-model of impact traumatic brain injury”	5 h	?	“Adult”
Martinez-Ramirez et al., 2022 [77]	“Robust, Long-Term Video EEG Monitoring in a Porcine Model of Post-Traumatic Epilepsy”	-	♂ (castrated)	20–26 weeks, 18–25 kg
O’Donnell et al., 2023 [21]	“Multimodal Neuromonitoring and Neurocritical Care in Swine to Enhance Translational Relevance in Brain Trauma Research”	36 h	♂	10–12 weeks, 25–30 kg
Datzmann et al., 2021 [20]	“In-depth characterization of a long-term, resuscitated model of acute subdural hematoma–induced brain injury”	54 h	♀/♂	9–18 months, 56–71 kg
Datzmann et al., 2022 [82]	“The effect of targeted hyperoxemia in a randomized controlled trial employing a long-term resuscitated, model of combined acute subdural hematoma and hemorrhagic shock in swine with coronary artery disease: An exploratory, hypothesis-generating study”	48 h	♀/♂ (castrated)	36–41 months, 56–71 kg
Datzmann et al., 2023 [83]	“An exploratory study investigating the effect of targeted hyperoxemia in a randomized controlled trial in a long-term resuscitated model of combined acute subdural hematoma and hemorrhagic shock in cardiovascular healthy pigs”	48 h	♀/♂ (castrated)	36–41 months, 56–71 kg

## Data Availability

No new data were created or analyzed in this study. Data sharing is not applicable to this article.

## Abbreviations

The following abbreviations are used in this manuscript:

ASDH	Acute subdural hematoma
CCI	Controlled cortical impact
CPP	Cerebral perfusion pressure
CSF	Cerebrospinal fluid
CT	Computed tomography
EEG	Electroencephalography
FPI	Fluid percussion injury
GFAP	Glial fibrillary acidic protein
ICP	Intracranial pressure
ICU	Intensive care unit
MAP	Mean arterial pressure
MGCS	Modified glasgow coma scale
MRI	Magnetic resonance imaging
PaCO_2_	Arterial partial pressure of carbon dioxide
PbtO_2_	Brain tissue oxygenation
TBI	Traumatic brain injury
UCH-L1	Ubiquitin Carboxy-Terminal Hydrolase L1
